# A Brief Overview of Radiation-Induced Effects on Spermatogenesis and Oncofertility

**DOI:** 10.3390/cancers14030805

**Published:** 2022-02-04

**Authors:** Hisanori Fukunaga, Akinari Yokoya, Kevin M. Prise

**Affiliations:** 1 Center for Environmental and Health Sciences, Hokkaido University, Sapporo 060-0812, Japan; 2 Institute for Quantum Life Science, National Institutes for Quantum Science and Technology, Ibaraki 319-1106, Japan; yokoya.akinari@qst.go.jp; 3 Graduate School of Science and Engineering, Ibaraki University, Ibaraki 310-8512, Japan; 4 Patrick G Johnstone Centre for Cancer Research, Queen’s University Belfast, Belfast BT9 7AE, UK; k.prise@qub.ac.uk

**Keywords:** adolescent/young adult patient, ethical, legal, social issue, microbeam radiotherapy, oncofertility, radiation, spermatogenesis, transgenerational effect

## Abstract

**Simple Summary:**

Spermatogenesis is one of the most important processes for the propagation of life; however, the testes’ ability to form sperm via this differentiation process is highly radiosensitive and easily impacted by exposure to environmental, occupational, or therapeutic radiation. Furthermore, the possibility that radiation effects on the gonads can be passed on from generation to generation should not be overlooked. This review focuses on the radiation-induced effects on spermatogenesis and the transgenerational effects. We also explore the potential of novel radiobiological approaches to improve male fertility preservation during radiotherapy.

**Abstract:**

The genotoxicity of radiation on germ cells may be passed on to the next generation, thus its elucidation is not only a scientific issue but also an ethical, legal, and social issue in modern society. In this article, we briefly overview the effects of radiation on spermatogenesis and its associated genotoxicity, including the latest findings in the field of radiobiology. The potential role of transgenerational effects is still poorly understood, and further research in this area is desirable. Furthermore, from the perspective of oncofertility, we discuss the historical background and clinical importance of preserving male fertility during radiation treatment and the potential of microbeam radiotherapy. We hope that this review will contribute to stimulating further discussions and investigations for therapies for pediatric and adolescent/young adult patients.

## 1. Introduction

**Figure 1:**
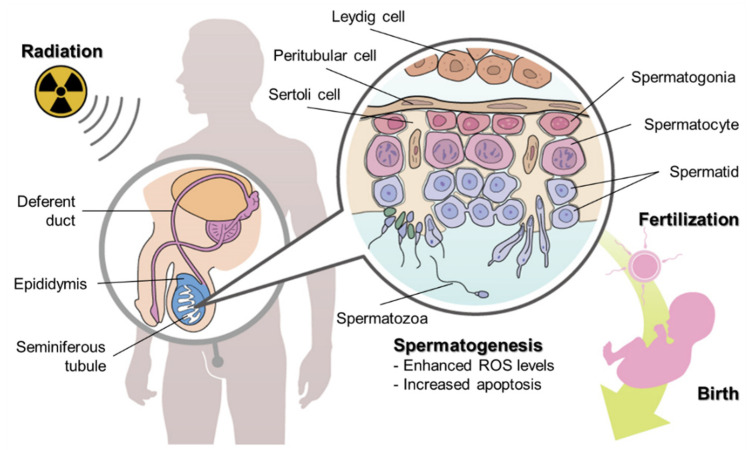
Irradiation, spermatogenesis, fertilisation, and birth. The radiation-induced impacts on germ cells during spermatogenesis may be passed on to the next generation. If spermatozoa (sperm) were to form in the seminiferous tubules after irradiation, they would be ejaculated through the epididymis and deferent duct, leading to fertilization.

Spermatogenesis is an essential physiological process for living organisms, in terms of the inheritance of the genome. This long and dynamic differentiation process in the seminiferous tubules takes about 60–70 days in humans and about 30 days in mice, which results in the transformation of spermatogonial stem cells (SSCs) through spermatogonia, primary spermatocytes, secondary spermatocytes, and spermatids to spermatozoa, which then mature in the epididymis. During the spermatogenesis process, radiation-induced effects can cause reproductive toxicity [1]. For high-dose exposures, this can be observed clinically, as there is a decrease in the fertility of cancer survivors following radiotherapy, especially among paediatric and adolescent/young adult (AYA) patients. In recent years, the concept of oncofertility, a combination term of oncology and fertility, has been proposed [2], and the importance of fertility preservation for cancer patients is now widely considered. In addition, the effects of occupational and environmental radiation exposure (i.e., chronic low-dose-rate (LDR) exposure) on the process of spermatogenesis and potential associated effects on the next generation are another issue of concern for modern society (Figure 1) [3]. The concerns today focus on LDR exposures received by not only members of the public and traditional industry workers, but also astronauts, as we enter a new age of space exploration [4]. Further investigations on the impact of these exposures will be required in the near future.

The first recognition of the possibility of radiation-induced genotoxicity came in 1903 when Heinrich Albers-Schönberg demonstrated that X-rays could damage rabbits’ testes [5]. Hermann Muller, the 1946 Nobel laureate in Physiology or Medicine, showed that X-rays could produce mutations in fruit flies and that the prevalence of the effect was approximately linearly proportional to the dose [6]. In addition, based on the accumulated knowledge of radiation epidemiological studies over a long period of time, including the Life Span Study of the atomic bomb survivors in Hiroshima and Nagasaki [7,8,9], it is now agreed that the cancer and non-cancer effects of radiation are dose-dependent, regardless of the presence or absence of a threshold [8,10,11].

In recent years, as the development of stem cell biology has deepened our understanding of the hierarchy of stem cell-centred tissues and the interactions between cells and their microenvironment, there has been a movement to re-evaluate the biological effects of radiation at the tissue level from the perspective of radiation biology [12]. In fact, according to *Publication 131*, of the International Commission on Radiological Protection, the behaviour of stem cells in living tissues, particularly the competition and selection among them for the microenvironment (niche) necessary for stem cells to maintain their properties (stem cell competition), may be involved in radiation effects at the tissue level [13]. The testes, the biological tissue responsible for spermatogenesis, have also received renewed attention to their characteristics, given their dynamic and complex systems of stem cells. Specifically, ‘non-targeted effects’ (i.e., interactions between cells directly hit by radiation tracks and those not hit by radiation tracks) are also thought to be involved in tissue-level radiobiological effects at low doses, such as environmental radiation exposure [14], and their elucidation is of importance for further understanding of radiation-induced spermatogenic impairment.

To clarify the radiation-induced effects on germ cells, it is also necessary to elucidate the potential role of transgenerational/genetic effects. However, these effects following paternal exposure are still controversial, probably because there appears to be a divergence between the results of animal experiments over the years and the results of epidemiological studies on humans, as discussed later in this review. In animal models, there have been several reports on the biological effects of radiation on the next generation; however, in the case of humans, previous studies, including epidemiological studies of people exposed to the atomic bombs in Hiroshima and Nagasaki, have shown no evidence of transgenerational effects of radiation. Genotoxicity in germ cells is not only a scientific issue but also an important ethical, legal, and social issue (ELSI). For example, it has become a major concern for victims of nuclear weapon fallout, victims of radiation accidents or nuclear disasters, and others who have suffered unexpected exposure to radiation. Therefore, from the perspectives of both radiobiology and epidemiology, future research on the transgenerational effects of radiation is required.

This review outlines our current knowledge on spermatogenic impairment associated with radiation exposure and the potential effects on the next generation, as well as the importance of fertility preservation in cancer treatment, highlighting the latest findings in radiobiology. We hope that the summation of past findings in this field will lead to the development of future research strategies and improve understanding of radiation exposures on spermatogenesis and its impact on oncofertility.

## 2. Radiobiological Perspective on Radiation-Induced Male Infertility

### 2.1. Radiation-Induced Impacts on Spermatogenesis

An experiment was conducted in the 1970s using prisoner volunteers who agreed to have their testicles irradiated to investigate the spermatogenetic effects of radiation in humans [15]. Participants who were exposed to a 0.11 gray (Gy) dose recorded a significant suppression of sperm count, and those exposed to doses of 3 to 5 Gy were rendered permanently sterile. In clinical settings, treatments using radiation may lead to temporary, long-term, or even permanent gonadal toxicity [16,17]. A review paper published in 2019 considered several previous studies and summarised the apparent sensitivity of gonadal tissues following a single dose of irradiation and time to recovery [18]. The time required for recovery increases with greater doses; complete recovery can be expected 9–18 months following radiation of <1 Gy, but for exposure greater than 2–6 Gy, permanent azoospermia in where there are no sperm in the ejaculate may be the result [18]. With these observations, radiation effects on male germ cells in gonadal tissue have been defined as tissue reactions (formerly called deterministic effects) [19], where the response is dose-dependent but also the severity has a threshold dose. It is also important to note that for cancer treatment, fractionated doses are commonly used which leads to greater delays in spermatogenic recovery. For example, a total gonadal dose of approximately 2.5 Gy, when delivered via fractionated radiation, can be expected to lead to permanent azoospermia [20]. This lower threshold dose for fractionated exposure, compared to a single-dose exposure, also known as ‘the reverse fractionation effect’, is one of the most intriguing elements among the radiation-induced impacts on spermatogenesis, in comparison to other organs. Furthermore, based on data revealed in recent studies, these thresholds appear to be significantly affected by the radiation dose rate. One previous study has demonstrated that LDR radiation leads to more adverse testicular damage than that following high-dose-rate (HDR) radiation, in contrast to the observed responses of other organs [21]. Research suggests that the DNA damage to spermatogonia cells and other cells during meiosis that is induced by chronic LDR irradiation may not be repaired efficiently, and the accumulating mutations may result in enhanced cell death when compared to damage induced by HDR radiation. Nevertheless, the underlying mechanisms of this process remain to be determined [21]. This characteristic, known as the ‘inverse dose-rate effect’, is another important radiation-induced impact on spermatogenesis.

### 2.2. Effects of Reactive Oxygen Species on Spermatogenesis

For many years, scientists have known that radiation can lead to a broad spectrum of DNA lesions, including damage to the nucleotide bases; DNA single and double-strand breaks (DSBs); cross-linking, and that exposure to radiation can seriously damage biological systems by triggering cell death or inducing mutations, which prompts radiation-induced carcinogenesis [22]. The different ways that biomolecules are affected by radiation can be classified broadly relative to the direct energy deposition and modifications via the creation of free radical molecules, such as reactive oxygen species (ROS). The latter can be found even at low doses [23]. ROS can be generated in cells by both exogenous and endogenous stimuli. The endogenous production of ROS mainly arises from leaks that form during mitochondrial electron transport chain activity [24]. ROS are generated in cells in equilibrium with a wide variety of antioxidant defences, but if the antioxidant detoxification systems fail to maintain low, tolerated levels of ROS, it can lead to excessive cellular levels of ROS that are deleterious and trigger oxidative stress [25].

ROS cause several forms of sperm DNA damage, including chromosome deletion, chromatin cross-linking, base oxidation, and DNA strand breaks [26]. ROS are also important apoptosis mediators by inducing cytochrome c and caspases 9 and 3; this results in a high frequency of single- and double-stranded DNA breaks [27]. Enhanced ROS levels in semen producing abnormal spermatozoa are a main cause of low fertility and even infertility; for example, teratozoospermic men with high ROS levels in their semen often demonstrate low fertility, if not infertility [28,29]. These data suggest the radiation-induced effects on spermatogenesis via elevated ROS levels in sperm-forming cells.

### 2.3. DNA Damage Repair Responses in Spermatogenic Cells

Spermatogenic cells consist of several subpopulations, including SSCs and their differentiating progeny (i.e., spermatogonia, spermatocytes, spermatids, and spermatozoa) (Figure 1), which have varying levels of radiation sensitivity [30]. Mitotically active cells such as the transit-amplifying and differentiating spermatogonia are most sensitive to radiation-induced killing. Furthermore, these differences may be due to the various cell types’ diverse chromatin compositions. As a result, they rely on different repair proteins and mechanisms to restore genomic integrity following radiation [30,31]. The failure to correctly repair these cells’ DNA damage leads to a de novo mutation in the germ cell line and a number of biological consequences [32].

One essential physiological mechanism that occurs in the testis is apoptosis, the process by which the number of germ cells in the seminiferous epithelium is limited [33]. Clinical studies have revealed that men with abnormal sperm parameters show higher levels of the apoptotic protein Fas in their ejaculated spermatozoa [34], indicating that apoptosis controls the selective depletion of abnormal spermatozoa. Phosphorylated histone H2AX (γ-H2AX) foci are generated in spermatogonia, spermatocytes, and round spermatids in response to radiation-induced DNA damage [35]. γ-H2AX plays a critical role in the recruitment of DNA repair factors and DNA damage-signalling proteins, including the tumour suppressor p53, which is a key protein with a central role in DNA damage-induced apoptosis. γ-H2AX interacts with p53 to induce spermatogonial apoptosis in irradiated spermatogonia [35]. One prior study indicated that even at very low doses (10 mGy), apoptosis can be induced in spermatogonia and lead to the arrest of spermatogenesis [36].

SSCs are characterised by a lack of compacted heterochromatin. DNA damage detection and signalling are mediated in these cells by the absence of the transducer complex γ-H2AX/MDC1, and radiation-induced DSBs are most often repaired via DNA-dependent protein kinase catalytic subunit (DNA-PKcs)-independent mechanisms [30]. In response to genotoxic insults, effective cell cycle checkpoints in the differentiating progeny, but not in SSCs themselves, eliminate damaged cells via apoptosis. This ensures that only genetically intact information is transmitted to subsequent generations [30].

At the onset of the first meiotic prophase, chromosomes receive Spo11-dependent DSBs that are repaired by homologous recombination (HR). This promotes crossing over and ensures homolog separation during the meiosis I division [37,38]. In contrast to HR, non-homologous end joining (NHEJ), the major DSB repair mechanism during the G1 cell cycle phase, is downregulated during the early meiotic prophase [38]. The classical DNA-PK-dependent NHEJ pathway involving the DNA-PKcs is recruited by the Ku70 and Ku80 proteins to the damaged site, and then both end-positioned Ku and DNA PKcs mediate the recruitment of XRCC4/DNA ligase IV complex [31]. NHEJ appears to aid the repair of radiation-induced and replication-dependent DNA damage in somatic testicular cells. It may also be required for the repair of persistent Spo11-dependent and radiation-induced DSBs in late spermatocytes [38]. However, studies using severe combined immune deficiency (SCID) mice with DNA-PKcs-deficient round spermatids have shown DSB repair kinetics that are almost identical to those of DNA-PKcs-proficient spermatids. This indicates that the slow and incomplete DSB repair in round spermatids is independent of the classical DNA-PK-dependent NHEJ [30,31].

### 2.4. Individual Variations in Radiotherapy-Related Spermatogenic Impairment

While candidate gene approaches have made some progress, they have, to date, been largely unsuccessful in identifying robust biomarkers of radiosensitivity at the individual level; this is due to the lack of an integrated understanding of the role of individual radiosensitivity in overall response [39]. Clinical observation data collected from cancer patients, who have experienced severe adverse effects, indicate that the normal tissue toxicity following radiotherapy apparently varies from patient to patient. The process of characterising this radiosensitivity is difficult, as the risk of developing a particular normal tissue reaction is dependent, to a great degree, on the target organ [40]. Furthermore, radiation research has shown that some DNA repair-related genes show tissue- and organ-specific expressions [41,42]. When considered as a whole, this indicates that radiosensitivity specificity is a significant limitation of the candidate gene approach.

The mismatch repair (MMR) pathway plays an important role in maintaining genomic integrity; meiotic recombination; and gametogenesis, and genetic polymorphisms in MMR genes have been determined to be involved in the aetiology of male infertility [43]. MutS protein homolog 5 (MSH5) and MSH4 proteins in the MMR pathway form a hetero-oligomeric structure, and the cells’ heterodimer, acts in meiotic recombination [44]. Previous epidemiological studies have statistically proven that the gene variant *MSH5* C85T (Pro29Ser) (rs2075789) is related to an increased risk of male sterility [45] and radiotherapy-related spermatogenic impairment [46]. Radiotherapy applied to Chinese testicular-germ-cell-tumour patients created significant differences between the pre- and post-treatment sperm count ratios, sperm morphology, and the DNA fragmentation index with the CT + TT genotypes, compared to those of the CC genotype (*p* < 0.05), although the underlying mechanism has yet to be determined [46]. In addition, as shown in Table 1 according to a global reference for human genetic variation released from the 1000 Genomes Project Phase 3 of the International Genome Sample Resource [47], the reference allele (C) frequency of the global, the African, the American, the East Asian, the European, and the South Asian populations were 0.8982, 0.9811, 0.8400, 0.8661, 0.8996, and 0.8590 respectively. The combination of data science and population-based biobank collections will lead to a better understanding of the genetic basis of radiotherapy-related adverse events.

Infertility is still an unfortunate adverse effect of most pelvic and systemic cancer therapies, including radiotherapy, as it impacts the quality of life of survivors during their pre-reproductive and reproductive years. For the improvement of clinical practice and health policy, such human genetic data is useful in deciding the future direction towards precision radiotherapy and radiation risk assessment in the world. The combination of population-based biobanking and genomics project has great potential to provide clinical information to help overcome or minimise radiotherapy-related adverse events including radiation-induced spermatogenic impairment. These findings may provide an important opportunity for the realisation of precision genetic medicine in the future [48,49].

## 3. Transgenerational Effects Following Paternal Exposure

### 3.1. Animal Studies

In February 1927, Gager and Blakeslee reported the chromosome and gene mutations in the offspring of exposing flowers of Dature to Radium-derived radiation [50]. Their peer-reviewed research article published in *Proceedings of the National Academy of Sciences* was evidence for the first time of gene mutations (via the use of ionizing radiation), almost six months prior to Muller’s *Science* paper [6]. Since then, several animal experiments have revealed the transgenerational effects of radiation. In 2000, a landmark murine study demonstrated that the transgenerational effects of paternal exposure extend to the germ line of unexposed first-generation offspring, manifested by increased instability among repeat-DNA sequences in the descendants of the exposed mice [51].

Both epigenetic and genomic pathways have been suggested in explanation of the transmissible effects of environmental contaminants, including sperm DNA mutations, the suppression of germ-cell apoptosis, genomic instability, and imprinting errors [52,53]. Several other animal studies have indicated evidence of the transgenerational epigenetic effects of paternal radiation exposure [53,54,55,56,57,58,59]. In fact, epidemiological studies have pointed to transgenerational genomic instability in bank voles (*Clethrionomys glareolus*) around Chernobyl and butterflies (*Zizeeria maha*) around Fukushima following the nuclear accidents [60,61].

### 3.2. Human Epidemiological Data

Hypotheses concerning the transgenerational effects of paternal exposures to radiation remain controversial in their relationship to humans. A study by Jacqueline Fabia and Truong Dam Thuy in 1974 reported that paternal occupational exposure to chemical substances can affect the integrity of spermatogenesis and, potentially, lead to the transmission of carcinogenic defects in these workers’ children [62]. In the 1980s, public concern prompted the British government to investigate an apparent hot spot of cancers among children living in the vicinity of the Sellafield nuclear power plant, in Cumbria. A population-based analysis revealed a high incidence of leukaemia and lymphoma among younger residents of Seascale, a community located roughly two miles from the Sellafield plant, compared to statistics found in national registries and the surrounding area [52,63]. In addition, a case-control study indicated that the children of fathers who were working at the plant at the time of their conception had a threefold-greater risk of developing leukaemia or non-Hodgkin’s lymphoma before the age of 25 [64]. This is in contrast to studies of Japanese atomic bomb survivors. No evidence has been found for increased cancer incidence among the children of fathers who were exposed to those blasts [65,66,67]. Moreover, there is a recent report that shows no genetic effects of radiation in people exposed to the 1986 Chernobyl nuclear accident [68]. Furthermore, in clinical practice, a Danish case-cohort study demonstrated that mutagenic chemotherapy and radiotherapy doses delivered to the gonads were not associated with genetic defects in the children of cancer survivors, although larger studies must be conducted to explore in greater depth the potential associations between high-dose pelvic irradiation and specific adverse pregnancy outcomes [69]. Additional studies are also needed to clarify whether or not there are human transgenerational effects from the exposed male germ line. If necessary, international scientific organisations, such as the United Nations Scientific Committee on the Effects of Atomic Radiation and the International Commission on Radiological Protection, should move quickly to establish appropriate protective guidelines and promote the development of relevant legal systems.

Interventionary studies involving animals or humans, and other studies that require ethical approval, must list the authority that provided approval and the corresponding ethical approval code.

## 4. Oncofertility and Microbeam Radiotherapy

### 4.1. Fertility Preservation in Patients after Radiotherapy

The degree of adverse events in parallel organs (an organ composed entirely of many subunits, each of which is thought to perform the same function in parallel) is thought to depend on the proportion of the volume of the subunit in the total organ that is irradiated with a dose that results in functional damage. Thus, dose-volume histogram analysis is commonly used as a countermeasure for adverse events associated with radiotherapy. However, as mentioned above, one defining characteristic of non-uniform exposures is that the observed dose–response relationships cannot be predicted from uniform exposures and then based on the standard radiobiological DNA damage and repair model [70]. Although advances in radiotherapy technology have recently made it possible to efficiently focus radiation doses on the planned target volume (PTV), a technology to completely irradiate only cancerous tissues has not yet been developed. Therefore, spatially heterogeneous dose distribution in the normal tissues surrounding the PTV may still occur, resulting in adverse events. In fact, testicular doses can be estimated with a standard deviation corresponding to 1–2% of the tumour dose, which is sufficient for the purpose of determining whether or not fertility is at risk by a planned treatment [71]. Although the endocrine regulatory system is more radioresistant than spermatogenic cells, high doses of radiation treatment can lead to infertility [1].

While recent improvements in treatment outcomes have resulted in fewer deaths among paediatric and AYA cancer patients, fertility preservation remains an increasingly important consideration in clinical oncology. Male patients, especially those with testicular or hematologic cancers or brain tumours, are more likely to present with infertility after being treated with some combination of chemotherapy and radiotherapy, thus affecting their quality of life. In addition, a previous study using marmoset models indicated radiation vulnerability in prepubertal testes [72]. In light of this situation, the concept of oncofertility has become widely popular in recent years [2]. In 2006, the American Society of Clinical Oncology (ASCO) and the American Society for Reproductive Medicine jointly published the first guidelines for fertility preservation in young cancer patients [73]. The guidelines emphasised that all cancer patients of reproductive age should be informed about the potential of infertility due to their cancer treatment, that oncologists should present fertility issues to patients early in the diagnosis process, and that collaborations with physicians specialising in reproductive medicine will be important. The ASCO guidelines were revised in 2013 and stated the need for all health providers involved in fertility preservation treatment to be responsive to patients [74]. The 2018 revision of the ASCO guidelines further pointed to the need to discuss fertility preservation as early as possible, although no specific changes were made regarding fertility preservation therapies [75].

One typical fertility preservation therapy is sperm cryopreservation. In 2016, Moss et al. proposed that ejaculated semen is the most effective fertility-preserving therapy for male cancer patients in the AYA generation and should be collected before the start of cancer treatment [76]. If ejaculated semen cannot be obtained, sperm retrieval is performed surgically (i.e., via testicular sperm extraction (TESE)). A previous study reported that a sample of frozen testicular tissue has been used to produce live offspring in experiments on mice, showing the clinical implications for male young patients with cancer who become infertile due to cancer treatments including radiotherapy [77]. Another study showed live offspring can be produced using the sperm developed in vivo from cryopreserved prepubertal monkey tissues by grafting [78]. To ensure quality decision-making, patients should be informed of not only the risks and benefits of these fertility preservations, including TESE, but also the costs; long-term outcomes, including psychological well-being; and be asked to sign an informed consent form [79]. Studies have shown that male AYA cancer survivors who received fertility consultations were more likely to have greater reproductive concerns than those who did not receive a fertility consultation [80]. Future radiation biology studies should contribute to the resolution of these clinical issues and the associated ELSIs.

### 4.2. Microbeam Radiotherapy as a Possible Approach for Oncofertility

Brinster and Zimmermann, working in 1994, were the first to perform a male mouse germ cell transplant, thus demonstrating that the dynamics of SSCs via seminiferous tubules are important for maintaining spermatogenesis [81]. They showed that the precise control of SSCs following radiotherapy is important for preserving fertility. Microbeam technology is one of the ways of facilitating such a high degree of control, as it is marked by the ability to deliver precise doses of radiation to selected individual cells, or even to targeted subcellular organelles [82]. At the tissue level, Slatkin et al. developed the concept of microbeam radiotherapy (MRT) in the 1990s, which calls for the use of parallel 50–150 keV micron-wide X-ray beams for potential therapeutic advantages [83]. This radiotherapy is characterised by the spatial and periodic alternation of microscopic dose distribution [84]. The key physical parameters defining these beams for MRT are the beam cross-sections, the inter-beam distance, the peak to valley dose ratio, and the valley doses delivered due to scattering [70]. It has been reported that MRT exposure leads to a tissue-sparing effect (TSE) which is observable in various organs and species [85,86,87,88,89,90,91]. The TSE refers to the development of radiation tolerance at the tissue level following exposure to spatially fractionated radiation [92].

Can a TSE following MRT occur during spermatogenesis? To answer this question, an ex vivo mouses testicular tissue culture [93], serving as an experimental spermatogenesis model, and a transgenic mouse model expressing the meiosis-specific biomarker Acr-GFP were employed [94,95] with the aim of investigating the radiation-induced impacts of non-uniform radiation fields on spermatogenesis. This experimental model was able to accurately reproduce radiation-induced male germ cell toxicity, including temporary infertility and permanent sterility [96]. Furthermore, using a monochromatic X-ray microbeam [97], this system demonstrated the potential for additional investigations at the organ level. In 2019, Fukunaga et al. were the first to report the TSE of spatially fractionated micro-slit beams for the preservation of spermatogenesis [98]. Their findings further indicated that the survival and potential migration steps of non-irradiated SSCs in irradiated testicular tissue are required for an effective TSE during spermatogenesis [92]. These may also occur in other systems.

In 2020, following up on their initial report [98], Fukunaga et al. showed that the TSE in irradiated testicular tissue was more effective when more SSCs survived following exposure to spatially fractionated radiation [99]. These findings support the view that the distribution of radiation delivered to the testicular tissues at the microscale level is important for preserving male fertility and indicate that stem cell migration/competition is, perhaps, involved in the TSE’s underlying mechanisms [92]. The testicular TSE of spatially fractionated X-ray irradiation has considerable potential for future clinical applications, as it responds to a wide range of X-ray energy, although further in vivo mechanistic studies are needed [100].

The results of a recent study on ultra-high dose rate (FLASH) radiotherapy demonstrated the remarkable sparing of normal tissue at this high rate of irradiation (>40 Gy/s), thus suggesting that the dose rate is also important for effective TSE [101,102,103,104]. It is true that MRT is most times synchrotron-generated and delivered in FLASH mode, but biological factors such as stem cell migration also contribute to the TSE of MRT based on spatial fractionation. The mechanisms underlying TSE remain ill-defined and further investigations using a variety of approaches are warranted [105]. Future radiobiological research of MRT has the potential to revolutionise the landscape of oncofertility.

## 5. Conclusions

Although several previous studies have elucidated the effects of radiation on spermatogenesis and transgenerational effects, many unresolved issues remain. The preservation of fertility following radiotherapy is of importance for paediatric and AYA cancer patients. Radiobiological research using microbeams and other novel techniques is expected to make significant contributions to this field.

## Figures and Tables

**Table 1:** Regional difference of the *MSH5* C85T variant frequency.

Population	Sample Size	Allele Frequency	
		Cs	T
Global	5008	0.8982	0.1018
African	1322	0.9811	0.0189
American	694	0.8400	0.1600
East Asian	1008	0.8661	0.1339
European	1006	0.8996	0.1004
South Asian	978	0.8590	0.1410
